# Supplementary material for: Quantitative In-Depth Transcriptome Analysis Implicates Peritoneal Macrophages as Important Players in the Complement and Coagulation Systems
Source: Int J Mol Sci. 2022 Jan 21;23(3):1185. doi: 10.3390/ijms23031185 (PMC8835655; doi:10.3390/ijms23031185)
Supplement: Supplementary file 1 [file ijms-23-01185-s001.zip › ijms-1528669-supplementary.pdf]

Supplementary Table S1. Human Monocyte samples

	Human -Monocytes-Ampliseq				
	Male Age 51	Male Age 43	Female Age 47	Male Age28	Female Age 61
**A. Amyloids and General Transcr**					
Saa3(serum amyloid, apolipoprotein)	0	0	0	0	0
Saa1 (serum amyloid, apolipoprotein)	0	0	0	0	0
Saa2 (serum amyloid, apolipoprotein)	0	0	0	0	0
Saa4 (serum amyloid, apolipoprotein)	0	0	0	0	0
Actb (beta actin)	19693	21775	32168	14866	23328
Tcn2 (Transcobalmin)	21	29	48	10	15
Tln1 (Talin 1 cytoskeletal memb. con.)	248	135	88	51	74
Itsn1 (Intersectin 1 membrane traf.)	0.1	0	0	0.2	0.6
Grn (Granulin)	588	689	2558	682	1041
Bst1 (ADP-ribosyl cyclase 2)	100	88	4.2	16	19
Gda (Guanine deaminase)	0	0	0	0	0
Hamp (Hepsidin Iron import)	0.7	0	0.3	1.7	0.1
Ninj1 (Ninjurin 1 apoptosis signal?)	578	413	229	240	323
Hal (Histidine amonia lyase)	9	16	39	31	33
Hdc (Histidine decarboxylase	0	0	0.1	0	0.1
Hrg (Histidine rich glycoprotein)	0	0	0	0	0
**B. Extracellular matrix**					
Fn1 (Fibronectin)	0	0.1	0	0	0
Prg4 (Proteoglycan 4, Lubricin)	0	0	0	0	0
Srgn (Serglycin)	3855	3641	4976	1310	5214
Sdc3 (Syndecan 3)	5	2	6	6	7
Ecm1 (Extracellular matrix prot.1)	1	0.5	2	0	1
**C. Anti-microbial proteins**					
Lyz1 (Lysozyme)	27394	29951	11290	25335	16400
Defb (Beta-defensins)	0	0	0.2	0	0.1
Camp (Cathelicidin)	1	1	2	3.	2
Cybb (Cytochr.b-245 (Nox2) Cytb558)	1081	1684	1423	677	867
Padi4 (Peptidyl arg. deim. type IV)	77	61	51	100	31
Flnb (Filamin B fagocytosis)	9	10	10	10	9
Flna (Filamin A)	1175	520	803	201	494
Timd4 (Binds Phosph. serine apopt. c.)	0	0	0	0	0
**D. Lipid mediators and metabolism**					
Alox15 (Arachinodate 15 lipoxygenase)	0	0	0	0.3	0
Pla2g7 (Phosplipase A2)	15	3	149	17	26
Alox5ap (Arach. 5 lipoxyg. act prot.)	111	81	95	85	85
Ptgis (Prosaglandin I syntase)	0	0	0	0	0
Alox5 (Arachinodate 5 lipoxygenase)	190	188	101	467	154
Dpep2 (Dipeptidase 2 memb. b.)	77	93	47	88	85
ApoE (Apolipoprotein E)	2	0.5	15	4	0.6
Pltp (Phospholipid transfer protein)	1	1	3	0.5	1

**Plin2** (Perilipin2 cytopl. lipid droplet b.)	777	250	762	91	718
**Retnlb** (Resistin like beta)	0	0	0	0	0
**Smpdl3a** (Sphingom. Pho.di. acid-l. 3)	3	4	5	1	5
**Lipn** (Lipase)	11	10	29	13	27
**E. Complement proteins and their receptors**					
**Cfp** (Compl. factor P, Properdin)	991	1017	1203	542	784
**C1qa** (Complement factor C1q A)	7	19	9	13	24
**C1qb** (Complement factor C1q B)	3	8	30	1	21
**C1qc** (Complement factor C1q C)	1	4	0.4	0.2	4
**C4b** (Complement factor 4B)	0.1	0.1	2	3	0.6
**C4a** (Complement factor 4A)	1	0.2	2	2	0.6
**Cfh** (Complement factor H)	0	0	0	0	0
**Fcna** (Ficolin A) (FCN1)	3198	2681	1284	1025	2183
**Vsig4** (Comp C3b rec.)	5	4	9	1	5
**C3** (Complement factor 3)	2	1	9	2	0.1
**CFB** (Complement factor B)	0.2	0.1	0.4	12	0
**C2** (Comlement factor 2)	14	9	7	8	12
**C3ar1** (C3a receptor)	41	23	48	4	14
**F. Coagulation proteins**					
**F5** (Coagulation factor V)	40	28	8	20	19
**F10** (Coagulation factor X)	0	0	0	0	0
**F7** (Coagulation factor VII)	0	0	0	0	0
**F12** (Coagulation factor XII)	1	1	0.4	6	0.4
**F9** (Coagulation factor IX)	0	0	0	0	0
**F2** (Thrombin)	0	0	0	0.3	0
**G. Proteases**					
**Mmp19** (Matrix Metalloprotease 19)	1	1	37	0.5	0.4
**Mmp9** (Matrix Metalloprotease 9)	9	1	50	11	0.4
**Mmp27** (Matrix Metalloprotease 27)	0	0	0	0	0
**Mmp12** (Matrix Metalloprotease 12)	0	0	0	0	0
**H. Protease Inhibitors**					
**Cst3** (Cystatin C)	3704	4034	2667	2446	4906
**SLPI** (Secretory leukocyte prot inh.)	0.4	0	1	0.2	0.6
**Serpinb2** (Serpin B2)	38	10	21	1	5
**Timp2** (Metaloproteinase Inhibitor 2)	283	198	195	233	205
**Timp1** (Metaloproteinase Inhibitor 1)	725	526	1871	153	533
**Serpinb9** (Serpin B9)	111	59	23	28	16
**I. Lysozomal proteins**					
**Laptm5** (Lysosomal memb. prot 5)	6180	2373	2042	682	2090
**Psap** (Prosaposin glycosphingolipids)	12296	11791	6862	4277	8850
**Man2b1** (Alpha-mannosidase)	5	4	1	1	2
**Ctsb** (Cathepsin B)	814	422	1399	56	311
**Ctsd** (Cathespsin D)	1171	1110	4241	876	1163
**Ctsl** (Cathepsin L) (CTSL1)	19	11	430	18	28
**Ctsa** (Cathepsin A)	192	129	254	217	236
**Ctss** (Cathepsin S)	5290	5923	455	1024	2853
**Ctsz** (Cathepsin Z)	1415	928	682	280	1151
**Ctsh** (Cathepsin H)	329	425	251	127	336

Ctsc (Cathepsin C)	145	172	29	62	118
Ctso (Cathepsin O)	9	14	3	3	11
Ctsf (Cathepsin F)	1	2	1	3	1.6
Ctse (Cathepsin E)	0	0	0	0	0
J Immunoglobulin receptors					
Fcgrt (FcRN)	380	243	65	129	104
Fcgr1A (Fc gamma receptor 1)	51	39	23	13	133
Fcgr2b (Fc gamma receptor 2B)	59	23	44	333	170
Fcgr2a (Fc gamma receptor 2A)	580	404	350	55	756
Fcer1g (Fc-epsilon receptor gamma)	1173	1426	1526	567	2096
K. MHC Class I and II					
B2m (beta-2 microglobulin)	5521	10399	4581	1885	11986
HLA-A	1548	1186	1598	420	383
HLA-B	6	8	3005	1780	39
HLA-C	2979	1265	3	403	5
HLA-E	2984	2412	3903	3178	3965
HLA-DRB1	3023	26	2126	1820	21
HLA-DRA	5490	7224	2592	934	6558
HLA-DPA1	2375	4872	2807	1656	5268
HLA-DPB1	1029	2162	978	551	1478
HLA-DPB2	0	0.4	0	0	0
HLA-DQB2	0	0	0	0	0.3
HLA-DQA1	103	6	285	86	9
HLA-DQA2	0	0.7	0	0	1
L. Classical surface receptors/markers					
CD14	1697	1633	1596	991	1525
CD40	6	11	25	10	19
CD28	1	0.7	1	0	0
CD86 (B7-2)	236	207	47	34	152
CD80 (B7-1)	2	0.4	14	2	1
CD83 (activation marker for dendritic c.)	18	19	362	44	39
CD244 (KIR2DL4)	31	49	70	63	53
CD84 (Ig superfamily)	20	51	128	26	32
CD209 (Rec. possibly inv. in phagoc.)	4	2.5	0.1	1	2
CD5l (CD5 like bind CD36)	0	0	0	0	0
Adgre1 (F4/80, Emr1, mucin like)	87	79	36	17	65
M. Scavenger receptors					
Marco (MARCO)	1	0.4	6	2	4
CD163 (Scavenger rec.)	225	169	778	37	173
CD36 (Scarb3) (Lung 1230)	250	392	241	405	315
CD68 (binds Oxidized LDL)	1273	1143	1536	546	564
CD177	0.3	0	1	0.5	0.1
Scara3 and 5	0	0	0	0	0
Scarb2	28	50	307	125	78
Scarb1	22	30	5	15	12
N. Cytokine, chemokine and endothelin receptors					
Fgfr1 (FGF receptor 1)	0.1	0	0.8	0	0
Csf1r (M-CSF receptor)	129	135	691	169	114

Csf2ra (GM-CSF receptor alpha chain)	47	53	362	100	74
Csf3r (G-CSF receptor CD114)	1236	900	1031	3129	939
Ccr5 (CCR-5 receptor)	2	3	37	1	1
Ccr1 (CCR-1 receptor)	34	53	30	18	17
Tnfrsf1b (TNF receptor rec. Subf. 1b)	1515	1100	780	267	1101
Tnfrsf1a (TNF receptor rec. Subf. 1a)	199	172	18	24	20
Tnfrsf11a(TNF receptor rec. Subf. 11a)	0.5	0.4	7	0.7	0.8
Tnfrsf21 (TNF receptor rec. Subf. 21)	25	3	30	0.7	7
Tnfrsf14 (TNF receptor rec. Subf. 14)	41	48	95	60	65
Il10ra (IL-10 receptor alpha)	609	443	237	357	422
Il15ra (IL-15 receptor alpha)	14	29	5	25	28
Il6r (IL-6 receptor alpha)	80	101	159	117	62
Il4r (IL-4 receptor alpha)	42	37	70	113	39
IL3ra (IL-3 receptor alpha)	8	9	57	9	20
Il13ra1 (Il-13 receptor alpha1)	123	122	10	15	46
IL21r (IL-21 receptor)	0	3	11	0.7	2
IL27ra (IL-27 receptor alpha)	32	38	101	27	26
Il2rg (IL-2 receptor gamma)	47	56	66	21	36
Il2rb (IL-2 receptor beta)	1	2	8	0.3	41
Il1r1 (Receptor 1 for IL1 alpha)	2	0.7	3	1	0.4
Ednrb (Endothelin B receptor)	0.2	0.4	6	0	0.1
**O. Toll Like Receptors and accessory proteins**					
Tlr4 (TLR-4)	24	44	197	25	20
Ly96 (MD2 LPS binding with TLR4)	8	10	0.2	5	6
Tlr7 (TLR-7)	9	26	7	2	12
Tlr1 (TLR-1)	6	27	22	11	10
Tlr8 (TLR-8)	46	137	40	26	155
Tlr2 (TLR-2)	40	47	299	12	34
Tlr3 (TLR-3)	0	0	0	0	0
Tlr6 (TLR-6)	2	2	9	2	0.7
Nlrc4 (Inflammasome related)	4	5	2	8	0.7
**P. Cell adhesion**					
Itgam (Integrin alpha m, CD11b)	245	216	82	304	139
Itga6 (Integrin alpha 6)	0	0.5	1	0.2	0
Itgb2 (Integrin beta 2)	1975	1423	862	685	1504
Itgb1(Integrin beta 1)	161	160	330	392	265
Itga4 (Integrin alpha 4)	230	331	44	144	408
Itgb7 (Integrin beta 7)	17	21	5	6	8
Itgav (Integrin alpha v)	2	10	119	11	6
Itga9 (Integrin alpha 9)	2	1	0.6	4	2
Itgb3 (Integrin beta 3)	3	4	1	0.7	1
Itgax (Integrin alpha x, CD11c)	249	216	673	126	140
Selp (P-selectin)	0	0.6	0	0.4	0.4
Emilin2 (Elastin microfibril loc. prot. 2)	162	218	404	213	369
Icam2 (ICAM 2)	39	52	73	3	38
Lgals3 (Galectin3, MAC2)	249	375	1533	109	414
**Q. Chemokines and Cytokines**					
Pf4 (Platelet factor 4)	3	4	3	5	3
Cxcl13 (B-cell attracting (BCA-1)	0	0	0	0.5	0
Ccl24 (Eotaxin-2 or MPIF-2)	1	0.6	36	0	0.1

**Cxcl16** (T-cell and NK cell attracting)	**307**	**134**	**972**	**55**	**432**
**Cxcl14** (attracting activated NK cells)	**0**	**0**	**0.4**	**0**	**0**
**Cxcl2** (also named MIP2 alpha)	**12**	**3**	**20**	**6**	**3**
**Cxcl1** (Neutrophil attr.(Gro-a or NAP-3))	**4**	**2**	**3**	**4**	**0.4**
**Cxcl12** (also named SDF1)	**0**	**0**	**0**	**0**	**0**
**Ccl5** (Rantes attr. T-cells, Eos and Baso.)	**8**	**7**	**13**	**4**	**15**
**Ccl11** (Eotaxin 1)	**0**	**0**	**0**	**0**	**0**
**Tgfb2** (TGF- beta 2)	**0.1**	**0**	**0.6**	**0**	**0**
**Tgfb1** (TGF- beta 1)	**918**	**555**	**1411**	**718**	**866**
**Il16** (IL-16)	**0**	**0**	**0**	**0**	**0**
**Csf1** (M-CSF)	**6**	**4**	**2**	**45**	**5**
**IL18** (IL-18)	**58**	**53**	**32**	**16**	**51**
**Il18bp** (IL-18 binding prot.)	**6**	**11**	**42**	**9**	**12**
**IL1a** (IL-1 alpha)	**0.2**	**0.1**	**26**	**0**	**0**
**Il15** (IL-15)	**6**	**22**	**4**	**19**	**16**
**IL27** (IL-27)	**7**	**5**	**3**	**11**	**12**
**Il13** (IL-13)	**0**	**0**	**0**	**0.5**	**0**
**Il12a & b** (IL-12a and b)	**1 & 0**	**1 & 0.1**	**0 & 0**	**6 & 0**	**1 & 0**
**Tnf** (TNF-alpha)	**131**	**91**	**228**	**72**	**86**
**Igf1** (IGF-1)	**0**	**0**	**0**	**0**	**0.1**
**Egf** (EGF)	**0**	**0.1**	**0**	**0**	**0**
**Pdgfa** (PDGF-A)	**0.4**	**0.4**	**5**	**0.5**	**0.6**
**Pdgfb** (PDGF-B)	**0.2**	**0**	**40**	**0**	**0**
**Vegfa** (VEGF-A)	**183**	**37**	**185**	**152**	**155**
**Vegfb** (VEGF-B)	**11**	**4**	**22**	**2**	**6**
**R. Signaling components**					
**Tyrobp** (TYRO protein kinase-bi. prot.)	**4617**	**4899**	**3882**	**727**	**4188**
**Dab2** (Disabled homolog 2)	**1**	**2**	**102**	**1**	**2**
**Pde2a** (cGMP-dep. cyclic phosphodi)	**5**	**3**	**23**	**1**	**4**
**Btk**	**50**	**63**	**25**	**15**	**29**
**S. Transcription factors**					
**Gata6** (GATA-6)	**0**	**0.7**	**0**	**0**	**0.1**
**Gata3**	**0.3**	**0**	**2**	**0**	**3**
**Gata2**	**0.3**	**0.4**	**0.4**	**0**	**0.3**
**Gata1**	**0**	**0**	**0**	**0**	**0**
**Mitf**	**4**	**11**	**37**	**4**	**5**
**Spi1** (Pu.1)	**1307**	**763**	**729**	**445**	**480**
**Myb**	**0.2**	**0.1**	**0**	**0**	**0**
**Runx1**	**42**	**22**	**12**	**56**	**11**
**Runx3**	**166**	**79**	**485**	**158**	**205**
**Creb3l1**	**0**	**0**	**0.1**	**0**	**0**
**Zeb2** (Zinc finger corepressor)	**242**	**302**	**396**	**377**	**321**
**Tox2**	**0.3**	**0**	**0.1**	**0**	**0.1**
**Ikzf1** (Ikaros, Zinc finger transcr. f)	**54**	**36**	**21**	**28**	**31**
**Foxp3**	**1**	**0.6**	**0.4**	**0.2**	**0.1**
